# Venovenous extracorporeal membrane oxygenation for severe exogenous lipoid pneumonia induced by diesel aspiration

**DOI:** 10.3389/fmed.2025.1641834

**Published:** 2025-10-15

**Authors:** Yunyi Ding, Zhongjun Zheng, Haizhen Wang, Wenqi Qi, Xiao Lu

**Affiliations:** ^1^Department of Emergency Medicine, Second Affiliated Hospital, Zhejiang University School of Medicine, Hangzhou, China; ^2^Key Laboratory of the Diagnosis and Treatment of Severe Trauma and Burn of Zhejiang Province, Hangzhou, China; ^3^Zhejiang Province Clinical Research Center for Emergency and Critical Care Medicine, Hangzhou, China; ^4^Research Institute of Emergency Medicine, Zhejiang University, Hangzhou, China; ^5^National Emergency Medical Rescue Base, Hangzhou, China

**Keywords:** diesel aspiration, venovenous extracorporeal membrane oxygenation, severe exogenous lipoid pneumonia, case report, critical care

## Abstract

This case report presents a novel application of venovenous extracorporeal membrane oxygenation (VV-ECMO) in the treatment of a patient with severe exogenous lipoid pneumonia caused by diesel aspiration. Although the patient ultimately died of septic shock, ECMO significantly improved lung function and the oxygenation index, demonstrating its potential as an innovative supportive treatment method for such severe respiratory diseases.

## 1 Introduction

Lipoid pneumonia is a rare disease caused by the accumulation of fat-like compounds of animal, vegetable, or mineral origin in the alveoli (1). It can be classified into endogenous lipoid pneumonia and exogenous lipoid pneumonia (ELP). ELP is a disease caused by the inhalation of mineral, vegetable, or animal oils and is extremely rare (2). Most treatments involve providing supportive therapy based on anti-inflammatory and anti-infectious measures, waiting for the condition to resolve spontaneously. The main reported treatment measures include discontinuing lipid exposure, intravenous corticosteroids (3), antibiotics, and therapeutic bronchoalveolar lavage (4). In a case of ELP also caused by diesel aspiration, a patient with milder symptoms was successfully treated with antibiotics, corticosteroids, and inhaled bronchodilators (5). Two pediatric patients aged approximately one year, who developed pneumonitis secondary to aspiration of oil-based cosmetics and organic makeup brush cleaner, respectively, achieved successful recovery with surfactant therapy combined with ECMO support (6, 7). However, in clinical practice, there remains a lack of generally recognized effective therapeutic approaches, nor is there a specific antidote analogous to surfactant that can be applied in adult patients. Additionally, ELP is often accompanied by respiratory tract infections (8). The Alshamran team reported a case of ELP accompanied by Mycobacterium infection that led to death (9). At the same time, more common complications of ELP include progressive respiratory symptoms, lung injury, and fibrosis (8). Due to the difficulty in treating these complications, ELP usually has characteristics such as a long onset time and poor prognosis.

Therefore, how to effectively treat ELP is an urgent problem to be solved. Extracorporeal membrane oxygenation (ECMO) is a blood-pumping machine that can relieve the load on the heart and lungs. It can be divided into veno-arterial extracorporeal membrane oxygenation (VA-ECMO) and veno-venous extracorporeal membrane oxygenation (VV-ECMO) according to the way the blood returns to the body. VA-ECMO is usually used for severe heart failure, while VV-ECMO is used for respiratory failure (10). This case report describes a patient with ELP who aspirated diesel, and we innovatively used VV-ECMO as a supportive therapy. It can not only support gas exchange and reduce the burden on the lungs but also facilitate subsequent treatments such as lung transplantation. Unfortunately, there is no clinical report of using VV-ECMO to treat ELP in adults.

The ethical review approval document for this case can be found in the Supplementary material.

## 2 A case

**Figure 1:**
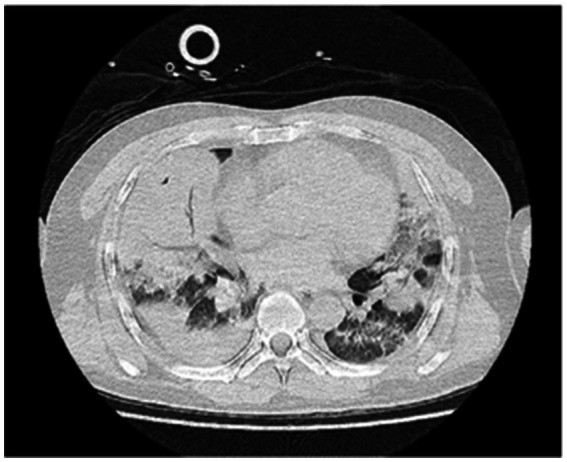
Lung CT scan upon admission.

A 48-year-old male was transferred to the emergency department of our hospital from another hospital due to “dyspnea for 4 days after diesel aspiration.” The patient aspirated approximately 50 mL of diesel 4 days ago and immediately developed a choking cough, chest tightness, chest pain, and dyspnea. Emergency chest CT showed extensive exudation and consolidation in both lungs, a small amount of right-sided pleural effusion with adjacent atelectasis of the lung tissue (Figure 1). Fiberoptic bronchoscopy revealed a small amount of yellow - brown thick sputum in the bilateral main bronchi, with mucosal congestion. There was also a small amount of yellow - brown thick sputum in the upper and lower lobes of both lungs, and the airway mucosa of the whole lung was congested, slightly edematous, with scattered bleeding points. The patient’s past medical history included an 8-year history of cerebral infarction and a patent foramen ovale occlusion surgery performed 8 years prior. The comprehensive diagnosis was “aspiration pneumonia (diesel).” Auxiliary examinations indicated ventilation dysfunction (SpO₂: 93%, PaO₂: 68.9 mmHg). The patient was intubated and connected to a ventilator for assisted ventilation and was admitted to the EICU for further treatment.

Four days ago, the patient was treated at a local hospital. Anti-infectious treatment with piperacillin-tazobactam combined with moxifloxacin was administered, along with anti-inflammatory treatment using methylprednisolone. Additionally, antitussive and expectorant treatments were provided as supportive measures. However, the patient’s condition did not improve. One day ago, the patient’s chest tightness and shortness of breath worsened, and the oxygen saturation dropped to 80%. Tracheal intubation was immediately performed. After ventilator-assisted ventilation, the patient was transferred to our hospital. Upon transfer to our hospital, to relieve the patient’s discomfort from using the ventilator, propofol and midazolam were used for sedation, and remifentanil was administered for analgesia. Meanwhile, piperacillin sodium-tazobactam sodium 4.5 g was continued as an intravenous drip every 8 h, combined with moxifloxacin 0.4 g given as an intravenous drip once daily for anti-infection (IL-6: 24 pg./mL, CRP: 111 mg/L). Methylprednisolone sodium succinate 40 mg was administered as an intravenous drip once daily for anti-inflammation. Additional treatments, including lung recruitment, prone position ventilation, early nutritional support, expectorant therapy, and other symptomatic supportive measures, were also implemented. Given the lack of improvement in the oxygenation index despite optimizing the ventilator parameters and continuous anti-infectious treatment, it was evident that alternative therapeutic strategies were needed. Considering that failure to maintain a normal oxygenation index could impact the prognosis, and on the premise of ensuring that the patient had no contraindications to VV-ECMO, VV-ECMO treatment was initiated on the 2nd day after admission. Catheters were placed in the right internal jugular vein (18F) and the right femoral vein (22F). The ECMO parameters were set as follows: rotation speed 3,500 r/min, blood flow 4 L/min, oxygen flow 6 L/min, and inspired oxygen concentration 100%. The ventilator parameters were: VT 200 mL; FiO₂ 90%; R 10 times/min; PEEP 10 cmH₂O. Subsequently, the patient’s oxygen saturation was maintained at around 90–95%.

A review of the medical history revealed that the oil barrel was severely contaminated. To preemptively address potential fungal infection, voriconazole, at a dosage of 200 mg administered every 12 h via nasogastric feeding, was initiated. On the 2nd day after VV-ECMO treatment, the patient’s oxygenation index continued to decline, and hemodynamic instability was noted. Given the significant increase in inflammatory biomarkers compared with baseline values (IL-6: 2251 pg./mL, CRP: 310 mg/L), along with the findings from the bedside chest X-ray, which revealed progression of exudative and consolidative changes in the left lung, and the results of flexible fiberoptic bronchoscopy and the bronchoalveolar lavage, which showed an increase in lung secretion compared with previous assessments, bronchoalveolar lavage was performed to detect potential respiratory tract infection. The culture results confirmed the presence of pan-drug-resistant *Acinetobacter baumannii* infection. Upon the treatment team’s assessment that the patient had developed septic shock, norepinephrine was administered to maintain hemodynamic stability. Given the confirmed pan-drug-resistant *Acinetobacter baumannii* infection and the patient’s septic shock, the antibiotic regimen was modified as follows: cefoperazone sodium-sulbactam sodium (2 g) was administered intravenously every 8 h; polymyxin E (150 mg, with an initial dose of 300 mg) was given intravenously every 12 h; and voriconazole (200 mg) was delivered via nasogastric feeding every 12 h. These adjustments aimed to effectively treat the patient’s drug-resistant *Acinetobacter baumannii* infection. The ventilator’s oxygen concentration was increased to 100%, while ECMO parameters were maintained. In addition, severe pulmonary edema and oliguria showed cumulative fluid overload. It posed a critical threat to the patient’s already compromised respiratory status. Therefore, we initiated CRRT.

**Figure 2:**
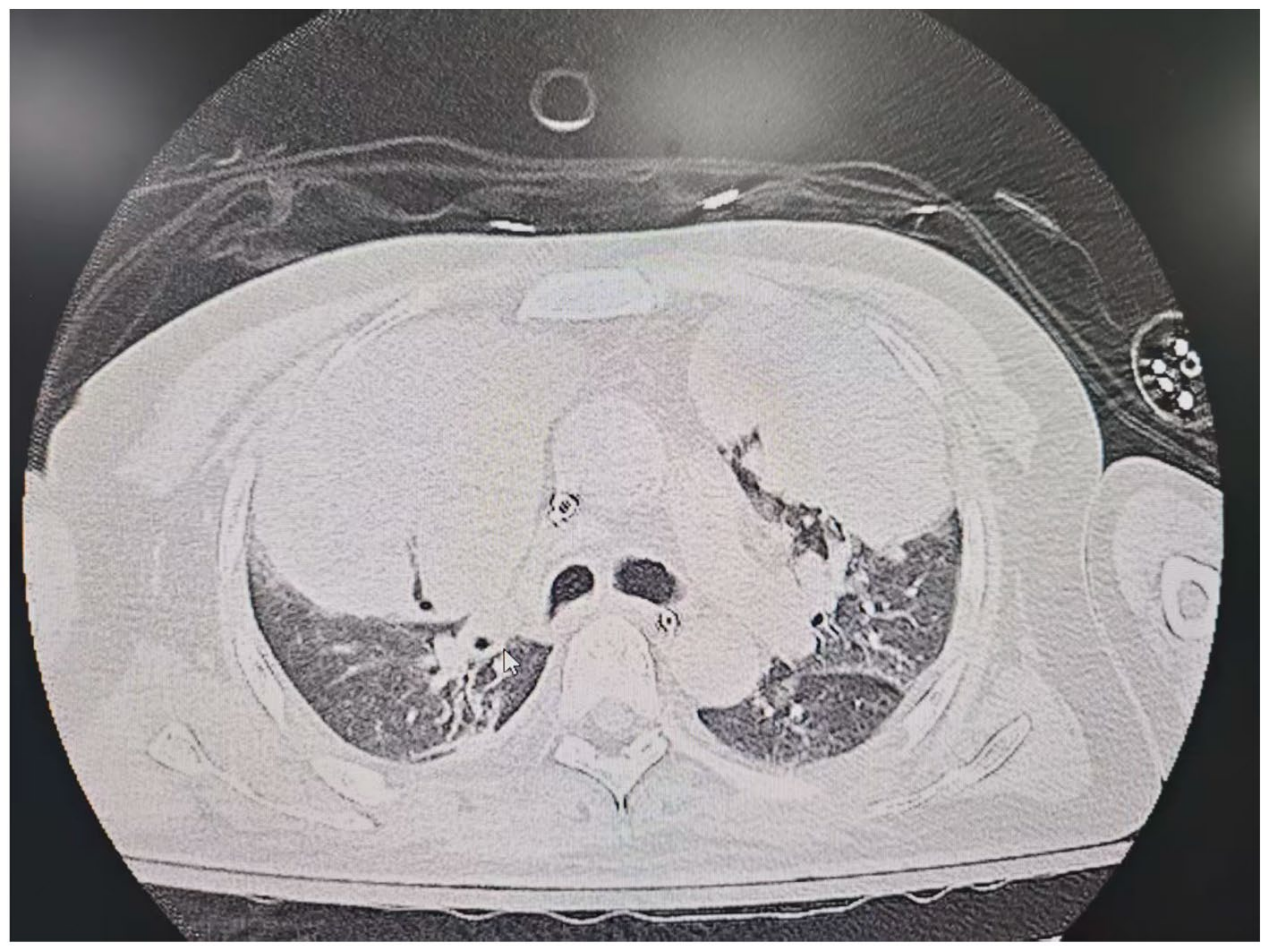
Lung CT on the day of ECMO weaning.

**Figure 3:**
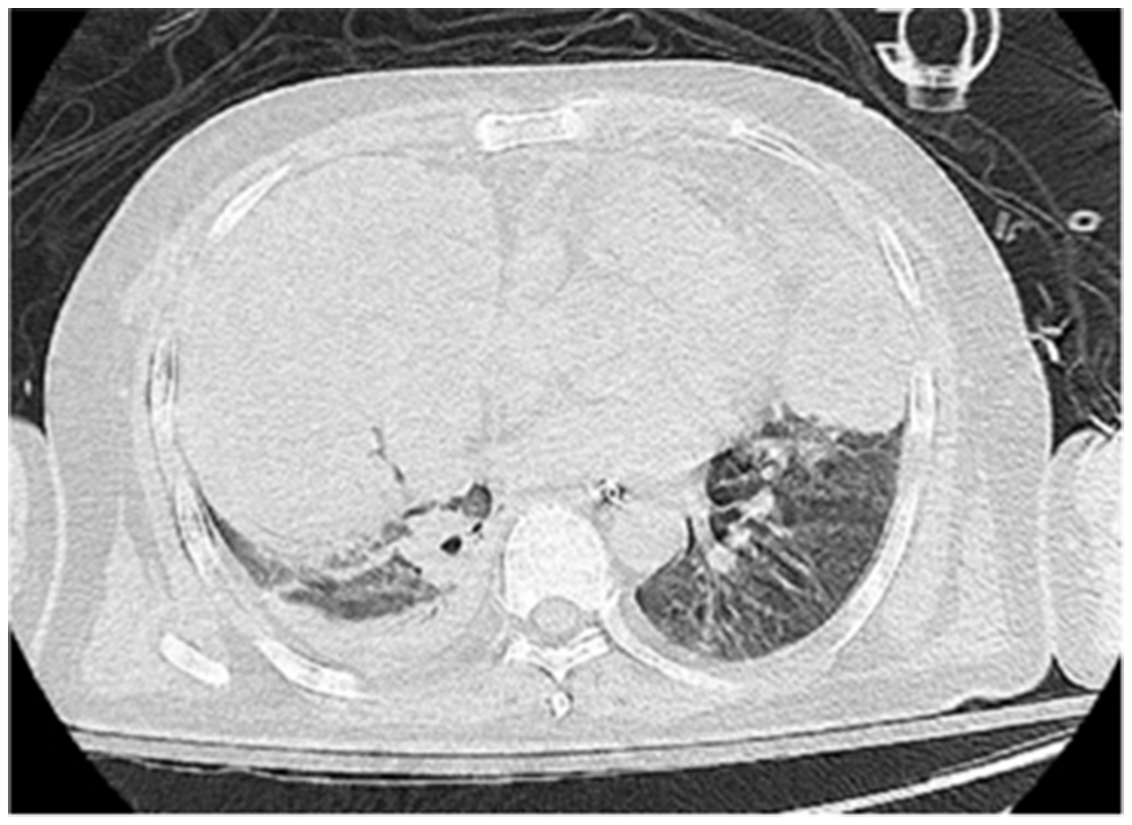
Lung CT scan on the fourth day after weaning from ECMO.

**Figure 4:**
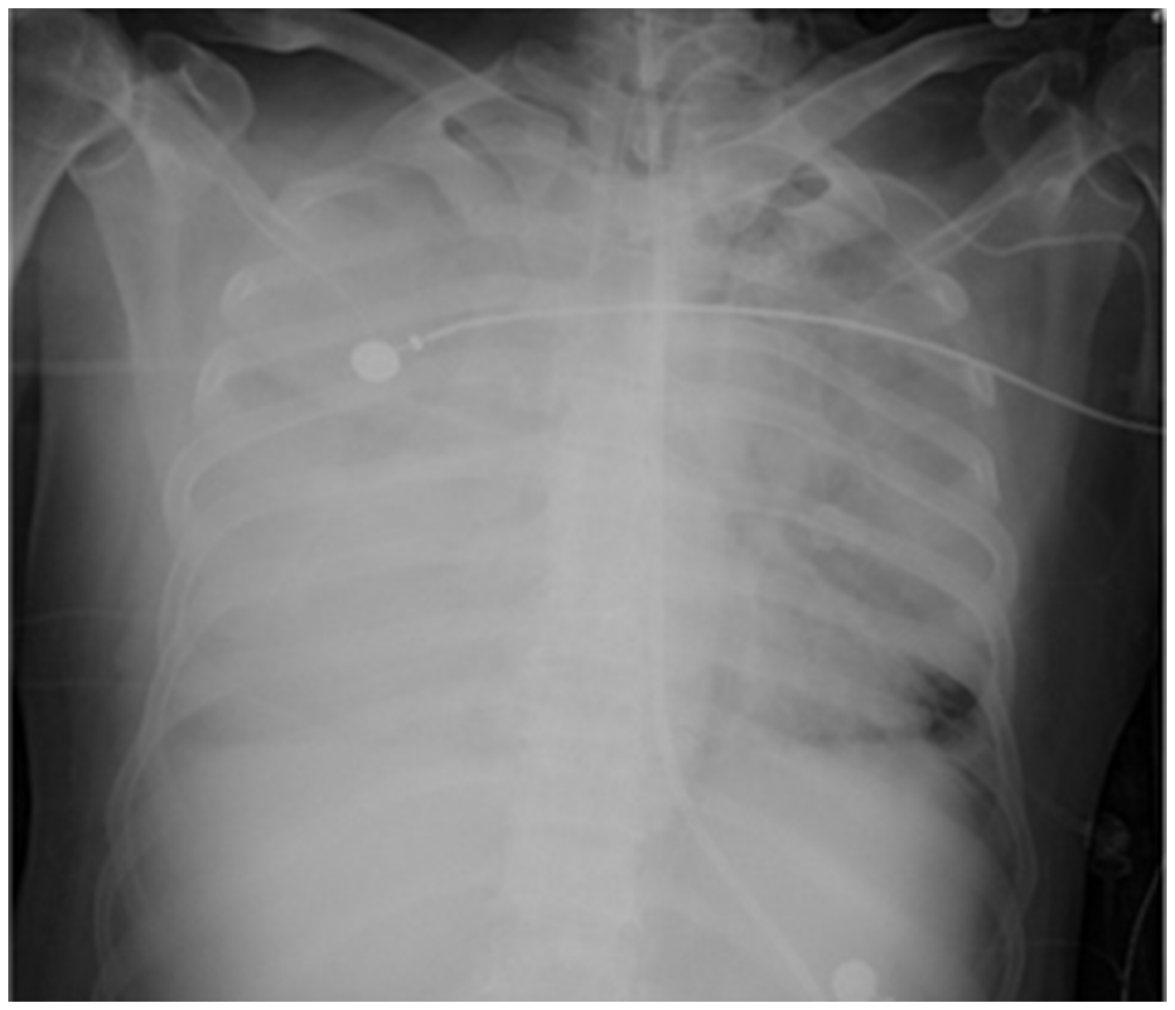
The last chest X ray scan on the seventh day after weaning from ECMO.

**Figure 5:**
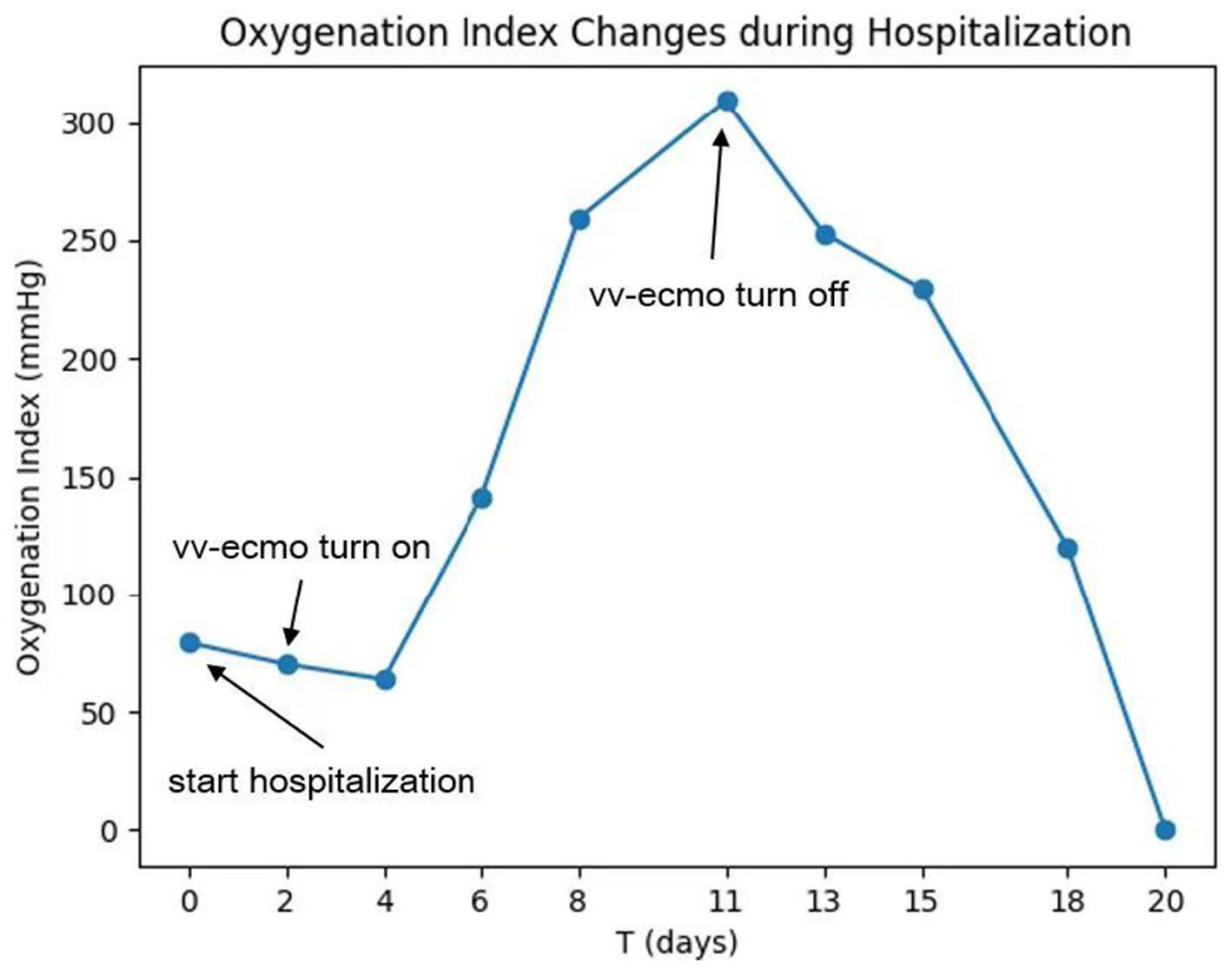
Oxygenation index fluctuation diagram.

**Figure 6:**
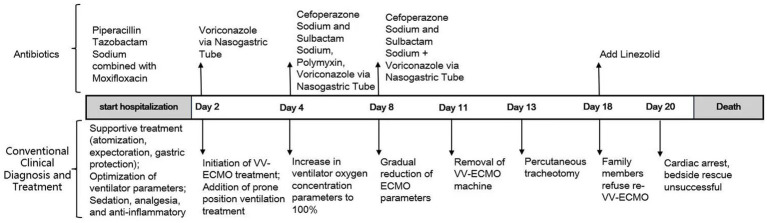
Treatment roadmap.

On the sixth day following VV-ECMO treatment, a favorable outcome was observed as the sputum culture yielded negative results (IL-6: 100 pg./mL, CRP: 195 mg/L), and there was a significant decrease in inflammatory indicators. The patient’s pulmonary condition demonstrated a marked improvement relative to the previous state, prompting a change in the antibiotic regimen to cefoperazone sodium-sulbactam sodium (2 g) administered every 8 h and voriconazole (200 mg) given via nasogastric feeding every 12 h. Concurrently, the patient’s oxygenation index improved, and lung function gradually recovered. This was based on the patient’s improved oxygenation and gradually recovering lung function, which indicated that the patient might be able to tolerate the discontinuation of ECMO support. The ECMO parameters were gradually titrated downwards, and the ECMO was weaned on the ninth day post-VV-ECMO treatment. At this point, the lung condition has improved significantly compared to before (Figure 2, and IL-6: 53 pg./mL, CRP: 202 mg/L). Given the patient’s persistent difficulty with extubation, the presence of retained respiratory secretions was suspected. Thus, percutaneous tracheotomy was performed on the second day post-ECMO weaning to alleviate the patient’s dyspnea symptoms. To further assess the patient’s lung condition following ECMO weaning, a repeat chest CT scan was performed on the fourth day after the procedure. The imaging revealed the emergence of new local masses with cavitation in the middle lobe of the right lung and the upper lobes of both lungs. This was suspected to be lipoid pneumonia with local lung tissue necrosis. Additionally, bilateral pleural effusion was noted, and the pneumonia had progressed compared with the previous state (Figure 3, and IL-6: 3557 pg./mL, CRP: 316 mg/L). Limited empyema was suspected, and the surgical department determined that the patient had indications for lung transplantation. However, the family members declined the surgery due to financial constraints. On the seventh day following ECMO weaning, the patient again exhibited hypotension, tachypnea, and tachycardia. Bedside chest X-ray suggested progression of exudation and consolidation in both lungs, along with bilateral pleural effusion (Figure 4, and IL-6: 5938 pg./mL, CRP: 322 mg/L). Septic shock secondary to pneumonia complicated by infection was suspected. Linezolid, at a dosage of 600 mg administered every 12 h, was added to the treatment regimen. Since the patient’s family members refused re-ECMO treatment, the deteriorating lung condition could not be reversed. On the ninth day post-ECMO weaning, the patient experienced cardiac arrest. Immediate bedside resuscitation was initiated. One minute later, the carotid artery pulsation ceased, and chest compression was terminated. The patient was declared clinically dead (The fluctuation of the patient’s oxygenation index can be observed in Figure 5, and the treatment route can be seen in Figure 6).

## 3 Discussion

We innovatively used VV-ECMO as a supportive measure in the treatment of a patient with acute ELP. VV-ECMO has shown great potential in the treatment of severe ELP, especially in patients with severe respiratory failure. It can not only provide respiratory support, but also gain crucial time for controlling the underlying condition and reducing subsequent lung damage. Unfortunately, the patient eventually died of septic shock after weaning from the machine.

This case reports an atypical case of acute ELP. ELP is usually uncommon (2). The earliest discovered ELP was caused by long - term use of laxatives and nasal drops (11). Subsequently, cases of ELP caused by various substances have been reported, including detergents (12), lipsticks, petrolatum (13), polyethylene glycol (14), and mineral oil (15). However, cases of ELP caused by diesel are extremely rare. The reason for ELP caused by diesel aspiration may be the minerals in diesel. These minerals are the key factors triggering lung inflammation. After entering the tracheobronchial tree, they can damage the mucociliary transport system, and once they enter the alveoli, they are difficult to excrete (16). Minerals cause the emulsification of oils, and the emulsified oils are absorbed by macrophages, but macrophages cannot metabolize these substances. When macrophages die, the oils are repeatedly released into the alveoli, leading to lesions such as cavities, chronic inflammation, and fibrosis (2, 17). In this case, the patient’s later lung CT showed the formation of alveolar cavities, which is consistent with the mentioned pathological mechanism.

In addition, a 2021 systematic review (8) summarized 90 ELP patients and found that the most common problems of this disease were infection with uncommon bacteria and respiratory failure. The patient in this case also had acute respiratory distress syndrome, which increased the difficulty of subsequent treatment and disease management. With the development of technology, people are increasingly exposed to hydrocarbons such as industrial emissions. However, there are few reports of ELP caused by hydrocarbons. A literature search found that there are a total of 5 case reports of ELP caused by hydrocarbons, including 3 cases of gasoline inhalation (18–20), 1 case of gas oil (21), and 1 case of diesel (22). Up to now, there has been no report of using VV-ECMO to treat ELP caused by diesel aspiration. For this patient, in addition to general basic symptomatic treatments such as antibiotics, the case innovatively added VV-ECMO treatment. This method can enable the patient to survive longer, potentially leading to recovery or allowing for further treatment decisions (such as lung transplantation). In this case, the ECMO was successfully weaned after 9 days of continuous treatment with VV-ECMO, improving the patient’s lung function. This proves that when VV-ECMO is applied to patients with severe ELP, it can provide key support for stabilizing the respiratory system. By bypassing the lungs, it can leave time for the lungs to heal, acting as a bridge to recovery. At the same time, it can also serve as a transition for future treatment options such as lung transplantation.

However, VV-ECMO also has limitations. First, as a supportive therapy, it cannot replace the therapeutic effect of symptomatic treatments such as specific antidotes on the disease, and there is currently no specific therapy for ELP caused by diesel aspiration. In addition, ECMO is only a temporary solution, and its long-term efficacy depends on the reversibility of the underlying lung injury. In severe ELP cases, if the recovery is not fast enough, patients may still die of organ failure or infection despite ECMO support. Finally, because ECMO requires continuous monitoring and professional care, it prolongs the patient’s ICU stay, increasing the risk of complications and the patient’s economic burden (23). However, it is undeniable that ECMO is still an effective method for critically ill patients with respiratory failure caused by severe lung diseases. A latest meta-report suggests that although ECMO has significant risks, compared with conventional management, the mortality rate of ECMO is reduced (RR 0.80, 95% CI 0.70 to 0.92; *p* = 0.002) (10). For infections complicated by ECMO, the current recommended strategy is to pay close attention during treatment to avoid infections (24). In this case, during the patient’s VV-ECMO period, we always closely monitored the infection situation and carefully adjusted the antibiotic use plan. The infection was once effectively controlled. Unfortunately, after the VV-ECMO was weaned, the patient unfortunately developed a secondary infection and died.

In addition, this patient was an auto repairman. During daily repair operations, due to potential safety hazards in the operation, he unfortunately aspirated diesel, which led to severe ELP. This case not only provides a reference for clinical treatment methods but also reveals, in a tragic way, the widespread but often overlooked risks in reality. We must attach great importance to and effectively strengthen safety education, especially in related industries such as auto repair. We should focus on standardizing the operation process and enhancing the safety awareness of practitioners to avoid similar health tragedies caused by diesel exposure as much as possible. Only in this way can we better protect people’s lives and health while pursuing economic development.

## 4 Conclusion

We used VV-ECMO for the first time to support the treatment of ELP. This case proves that the combination of VV-ECMO and other supportive measures can improve ELP to a certain extent. However, infections during and after the withdrawal of VV- ECMO are still severe challenges. Therefore, for such patients, early identification and active management of infection sources and complications are crucial. In addition, we call for strengthening safety education and preventive measures to avoid such accidents.

## Data Availability

The original contributions presented in the study are included in the article/supplementary material, further inquiries can be directed to the corresponding author.
